# Phytochemical Profiling and Antiviral Activity of Green Sustainable Nanoparticles Derived from *Maesa indica* (Roxb.) Sweet against Human Coronavirus 229E

**DOI:** 10.3390/plants12152813

**Published:** 2023-07-29

**Authors:** Fatma Alzahra M. Abdelgawad, Seham S. El-Hawary, Essam M. Abd El-Kader, Saad Ali Alshehri, Mohamed Abdelaaty Rabeh, Aliaa E. M. K. El-Mosallamy, Mohamed A. El Raey, Rania A. El Gedaily

**Affiliations:** 1 Department of Pharmacognosy, Faculty of Pharmacy, Heliopolis University, Cairo 11785, Egypt; fatma.mahrous@hu.edu.eg; 2 Department of Pharmacognosy, Faculty of Pharmacy, Cairo University, Giza 11562, Egypt; seham.elhawary@yahoo.com; 3 Department of Timber Trees Research, Horticultural Research Institute (ARC), Giza 12619, Egypt; eltorifi_ola@yahoo.com; 4 Department of Pharmacognosy, College of Pharmacy, King Khalid University, Abha 62251, Saudi Arabia; salshhri@kku.edu.sa (S.A.A.); mrabeh@kku.edu.sa (M.A.R.); 5 Department of Pharmacology, Medical Division, National Research Centre, Cairo 12622, Egypt; aliaamoneer@hotmail.com; 6 Department of Phytochemistry and Plant Systematics, Pharmaceutical Division, National Research Centre, 33 El Bohouth Street, Cairo 12622, Egypt; elraiy@gmail.com

**Keywords:** sustainable 1, LC-MS2, MRM 3, green synthesis 4, *Maesa indica* 5, coronavirus 6

## Abstract

Plant secondary metabolites are key components for new, safe and effective drugs. Ethanolic extract of *Maesa indica* Roxb. Sweet (ME) aerial parts were used for biosynthesis of sustainable green zinc oxide nanoparticles (ZnO NPs) with an average particle size 6.80 ± 1.47 nm and zeta potential −19.7 mV. Both transmission electron microscopy and X-ray diffraction assay confirmed the hexagonal shape of ZnO NPs. Phenolic ingredients in ME were identified using LC-ESI-MS/MS-MRM revealing the identification of chlorogenic acid, gallic acid, caffeic acid, rutin, coumaric acid, vanillin, naringenin, quercetin, ellagic acid, 3.4-dihydroxybenzoic acid, methyl gallate, kaempferol, ferulic acid, syringic acid, and luteolin. The major compound was chlorogenic acid at concentration of 1803.84 μg/g. The antiviral activity of ME, ZnO NPs, and combination of ME with ZnO NPs against coronavirus 229E were investigated. ZnO NPs had superior antiviral effect against coronavirus 229E than ME while their combination showed the highest anti-coronavirus 229E effect, with 50% inhibition concentration (IC_50_) of 5.23 ± 0.18 µg/mL and 50% cytotoxic concentration (CC_50_) of 138.49 ± 0.26 µg/mL while the selectivity index (SI) was 26.47. The current study highlighted the possible novel anti-coronavirus 229E activity of green ZnO NPs synthesized from *Maesa indica*. More studies are needed to further investigate this antiviral activity to be utilized in future biomedical and environmental applications.

## 1. Introduction

Environmental sustainability has become a major topic of concern all over the world; therefore, it should be taken into consideration during the search for new medications. Sustainability is how we meet our own demands without compromising the ability of future generations to meet their own demands [1]. The increasing need of inorganic nanoparticles (NPs) for many applications motivates the development of green synthesis pathways for nanoparticles by different plant extracts. Nowadays, green biosynthesis of nanoparticles has become important in large-scale production of medicines because of the method’s sustainability. The green formulation of nanoparticle has the advantage of being a rapid method to produce nano-crystals with different sizes and shapes [2]. Chemical synthesis of ZnO NPs uses unsafe hazardous and expensive chemicals while ecofriendly green synthesis of ZnO NPs uses plant extracts, which are safe and cheap; therefore, they are considered a safe, effective, and economic method [2]. 

In this study, we focused on the green synthesis potential of *Maessa indica* Roxb. A. DC. (Family Primulaceae), which is an evergreen, long, and smooth shrub known as wild berry or wild tea. It is native to Southern India and China [3]. It is also distributed in evergreen, semi-evergreen, and high humidity forests. This plant has many phytochemicals including flavonoids, phenolics, saponins, tannins, carbohydrates, fixed oil, and glycosides [4]. It contains numerous therapeutically active compounds, such as palmitic acid, chrysophanol, glyceryl palmitate, stigmasterol, β-sitosterol, dodecane, maesaquinone, quercetin 3-rhaminoside, rutin, chlorogenic acid, catechin, quercetin, nitrendipine, 2,3-dihydroxypropyl octadeca-9,12-dienoate kiritiquinon, and β-thujone [4,5].

Green synthesized silver NPs of *Maesa calophylla* Pit. and *Maesa laxiflora* Pit. Methanolic extracts have been reported to possess DPPH radical scavenging activity, reducing power and anticancer activity on adenocarcinoma human alveolar basal epithelial (A549) and cervical cancer (HeLa) cell lines mostly caused by necrosis and slightly caused by late apoptosis [6]. These results suggest their possible therapeutic effect in cancer. In virology, many metals and metal oxide nanoparticles are commonly used for different purposes, such as diagnosis, imaging process, prophylactic, and supplements for health improvement. They can be also used as therapeutic antiviral medications against different viruses, such as human rotavirus, hepatitis (A, B, C, and D), Japanese encephalitis, influenza, herpes simplex (1 and 2), human immunodeficiency virus (HIV), and human papilloma viruses. Sometimes these nanoparticles can be used to synergize the effect of antiviral medication [7,8]. ZnO NPs are approved as safe substances for humans’ medication by the American FDA (Food and Drug Administration). ZnO nanoparticles are cheaper than other metal oxide or metal nanoparticles. In addition, numerous studies have reported their antibacterial (Gram-positive and Gram-negative), anticancer, anti-inflammatory, antiviral, antidiabetic, antileishmanial, antioxidant, and wound healing activities [9].

Seven types of infectious human coronaviruses were recognized (Middle East respiratory syndrome coronavirus, severe acute respiratory syndrome coronavirus, severe acute respiratory syndrome coronavirus 2, OC43, NL63, 229E, and HKU1) [10]. Human coronavirus 229E is one of the major viruses responsible for upper respiratory tract disorders [10]. Coronavirus 229E is reported to be 65.04% identical to SARS-CoV-2 [11]. The coronavirus SARS-CoV-2 (COVID-19) is a novel enveloped, single-stranded large positive RNA virus that appeared in December 2019 [12]. It can infect humans, and it has caused a pandemic crisis all over the world. This virus originated from bats, and it is 96% matching with the bats’ coronavirus [13]. The disease usual symptoms are cough, fever, breathlessness, sore throat, fatigue, malaise, and loss of smelling and tasting sensation [14]. In most cases, the disease is mild, and sometimes, no certain symptoms were observed in patients, but in old people and people with health complications or comorbidities like cardiovascular, diabetic, and cancer patients, it may progress to pneumonia, acute respiratory distress syndrome, or multiple organ failure. The preventive measurements include enforcing public health and infection control measurements and vaccination [15]. 

To our knowledge, a few studies have reported the antiviral activity of ZnO NPs, especially anti-coronaviruses. For example, the antiviral activity of ZnO nanoparticles was reported against many viruses like influenza [16], hepatitis E and hepatitis C [17], rhinovirus [18], herpes viruses [19], human immunodeficiency virus (HIV), and finally SARS-CoV-2 [20]. The current study has investigated the anti-coronavirus 229E of ME and ZnO NPs as well as the ZnO nanoparticles loaded with ME. In addition, we aimed to identify polyphenolic compounds in ME using LC-ESI-MS/MS analysis. Triple quadrupole mass spectrometer detector was adapted in MRM (multiple reaction monitoring) screening mode. This method is characterized by its selectivity and sensitivity; therefore, it is suitable for the analysis of plant extracts and can differentiate between many compounds having the same parent ions, but they differ in the fragment ions [21].

## 2. Results and Discussion

### 2.1. Characterization of ZnO Nanoparticles

#### 2.1.1. FT-IR Analysis of Nano-ZnO and ME

**Figure 1:**
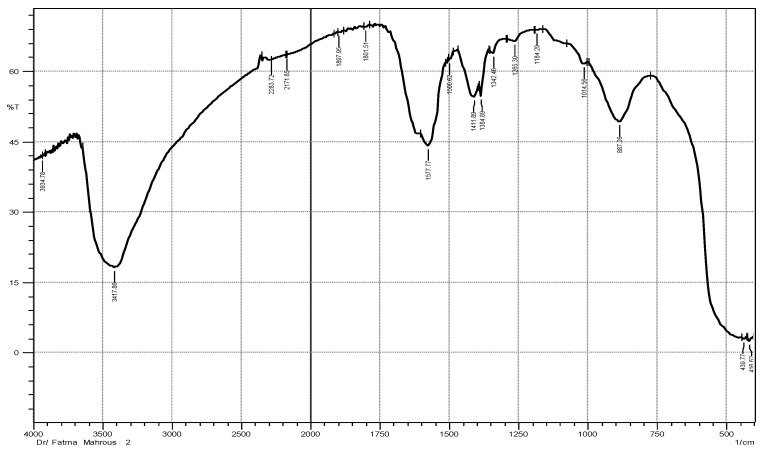
FTIR spectrum of ZnO NPs formed by *Maesa indica* 70% ethanolic extract.

**Figure 2:**
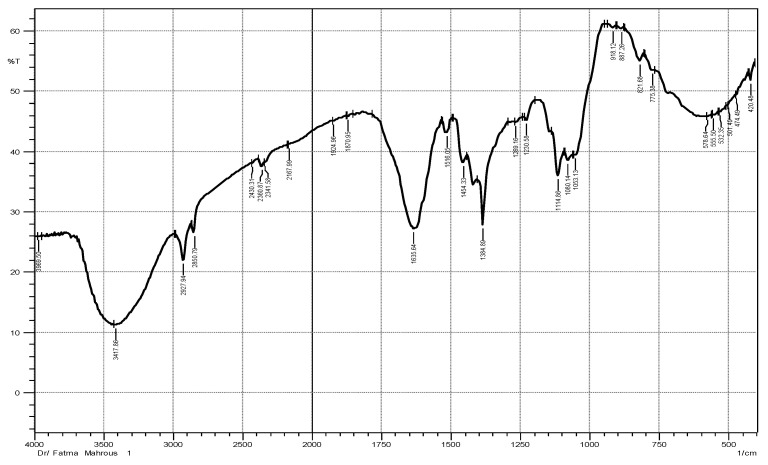
FTIR spectrum of 70% ethanolic extract of the of *Maesa indica*.

To detect the different functional groups in both ZnO NPs and ME, FTIR spectra for both were examined in the range 400–4000 cm^−1^. The spectrum of ZnO NPs is represented in Figure 1 showing different bands. According to this spectrum, a band at 3417.86 cm^−1^ (OH) was obtained due to water adsorption on ZnO NPs surface. Absorption bands at 1897.96 cm^−1^ and 1801.51 cm^−1^ indicated C=O stretching of the carboxylic group. The bands in the lower frequency regions (400–1400 cm^−1^) are characteristic to the compound like a fingerprint to a human. Absorption regions from 400 to 600 cm^−1^ were characteristic to metal oxides. The appearance of new bands at 439.77 and 416.62 cm^−1^ corresponded to the ZnO NPs. FTIR spectrum of ME is represented in Figure 2 showing bands different from ZnO NPs spectrum bands. The band at 3417.86 cm^−1^ was due to O-H stretching of alcoholic compounds. The bands at 2927.94 cm^−1^ and 2860.79 cm^−1^ were attributed to O-H stretching of carboxylic acid compounds. The bands at 1924.96 cm^−1^ and 1870.95 cm^−1^ indicated the C-H bending of aromatic compound. The bands at 918.12 cm^−1^, 887.265 cm^−1^, 821.68 cm^−1^, and 775.38 cm^−1^ were due to =CH bending of an alkene group [22,23]. All FTIR spectrum bands of ME and ZnO NPs as well as their corresponding functional groups were represented in Table 1.

These findings agree with previous studies on phytochemical screening of aerial parts of *M. indica*. In addition, the leaf extracts of *M. indica* were reported to contain phenolic compounds, flavonoids, saponins, tannins, and alkaloids, and IR bands of these compounds justified their presence [4,22].

#### 2.1.2. Zeta Potential ZnO NPs and DLS

**Figure 3:**
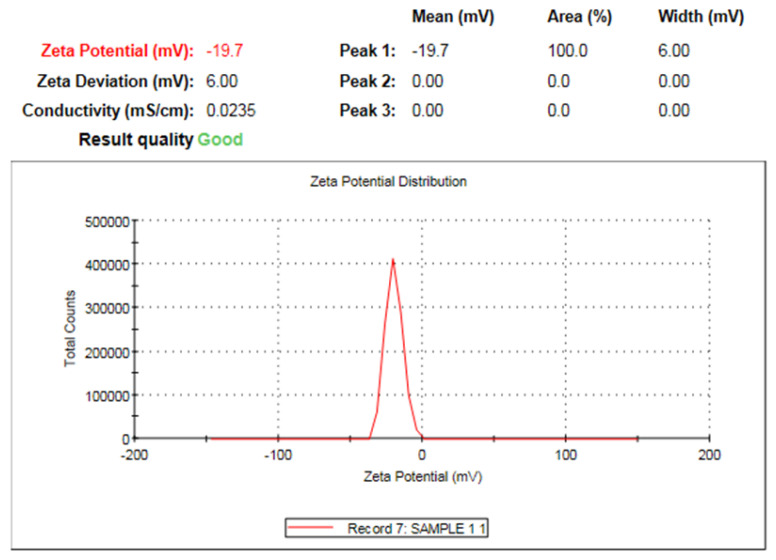
Zeta potential of ZnO NPs formed by *Maesa indica* 70% ethanolic extract.

The zeta potential of nano-ZnO is illustrated in Figure 3. The zeta potential peak of ZnO NPs shown at −19.7 mV confirmed the green synthesis of negatively charged nano-ZnO particles. The ZnO NPs were distributed in the medium carrying negative charge; the negative value of the zeta potential demonstrates the stability of nano-ZnO particles.

**Figure 4:**
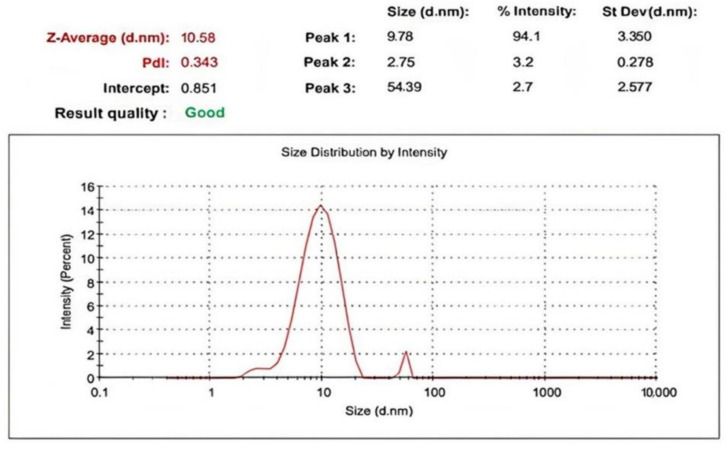
Zeta size of ZnO NPs formed by *Maesa indica* 70% ethanolic extract.

The nano-ZnO particles DLS (size-distribution image) is shown in Figure 4. The average particle size distribution of ZnO NPs varied from 3.350 to 10.58 nm while the PDI value was 0.343. The determined distribution of ZnO NPs size was between 2 and 68 nm.

#### 2.1.3. UV Analysis

**Figure 5:**
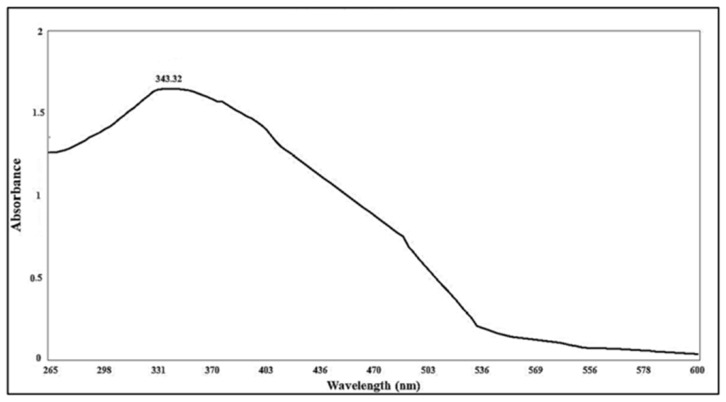
UV spectrum of nano-ZnO particles synthetized by 70% ethanolic extract of *Maesa indica*.

The UV assay of green synthetized nano-ZnO produced by ME had its maximum absorption peak at 343.32 nm. ZnO nanoparticles formation was confirmed by the absorption peak Figure 5 [24], as nano-ZnO had an absorption peak at a shorter wavelengths than the peak of typical ZnO. This coincided with the results of previous research that confirmed shorter wavelengths of nano-ZnO from typical ZnO. 

#### 2.1.4. Transmission Electron Microscopy (TEM) 

**Figure 6:**
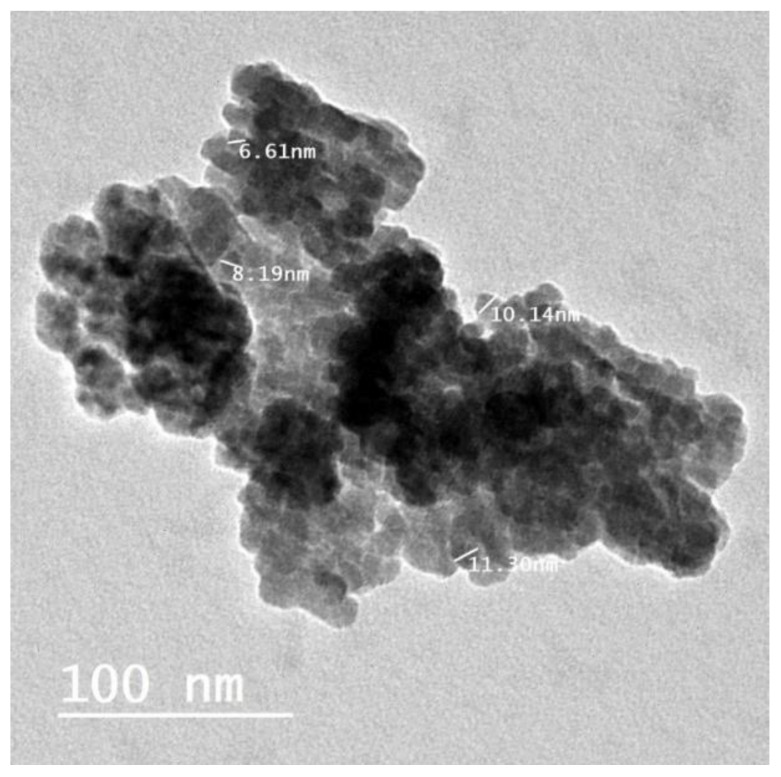
TEM analysis of ZnO NPs formed by *Maesa indica* 70% ethanolic extract.

Nano-ZnO particles were examined using both low- and the high-resolution power TEM examination. The result confirmed the hexagonal shape of nano-ZnO with a particle size ranging from 6.61 nm to 11.30 nm (the particle size average was 6.5 ± 1.47 nm) (Figure 6).

#### 2.1.5. XRD Analysis

**Figure 7:**
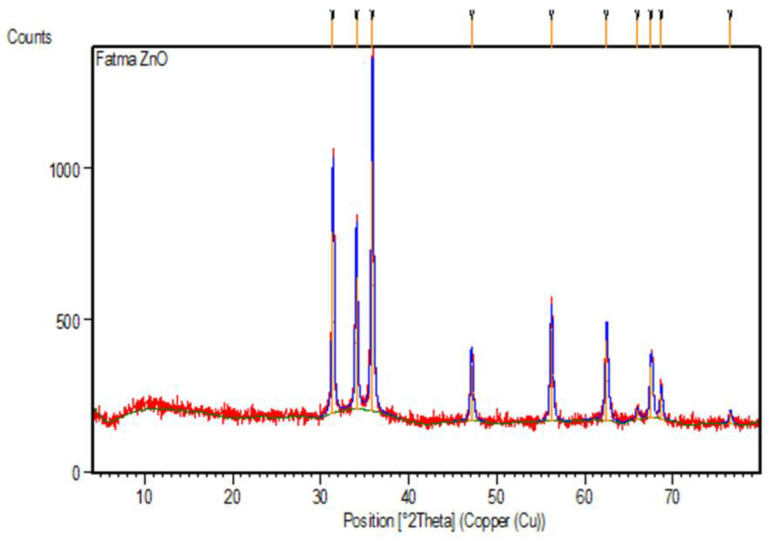
XRD analysis of ZnO NPs formed by *Maesa indica* 70% ethanolic extract.

X-ray diffraction (XRD) was used to confirm the presence of nano-ZnO and characterize their structure. Nano-ZnO particles bio-synthesized by ME showed 10 peaks with 2Ɵ values occurred at 31.403, 34.067, 35.881, 47.155, 56.218, 62.487, 65.98, 67.556, 68.68, and 76.55 corresponding to (100), (002), (101), (102), (110), (103), (200), (112), (201), (004), (202), and (203), respectively, Figure 7. There were no peaks other than the ZnO powder peaks in XRD confirming that the nano-ZnO was pure powder [25].

All the peaks were represented in a hexagonal phase crystal system with a reference code of (01-089-0510) and space group of P63mc. The crystal size was measured, and the computed average size was 0.519 nm. The equation that used to calculate the crystal particle size was Scherrer’s equation.

D = Kλ/β cosθ

D: Crystal Size 

K: Scherrer constant = 0.9

λ: X-ray Wavelength of the used beam (1.5406 Å).

β: Full width at half maximum

θ: Is the Bragg angle

### 2.2. LC-ESI-MS/MS-MRM Analyses

**Figure 8:**
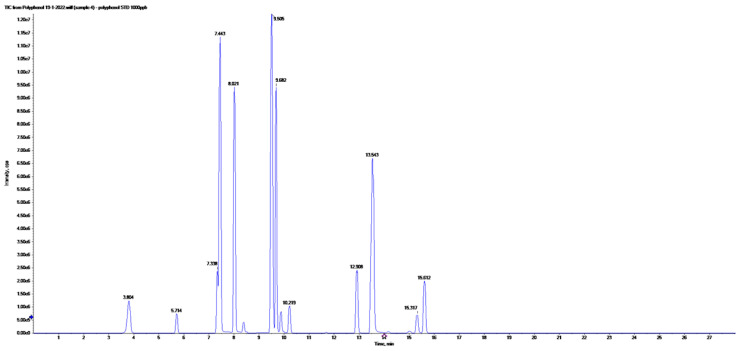
The LC-ESI-MS/MS chromatograms obtained in MRM mode of standard phenolic and flavonoids.

**Figure 9:**
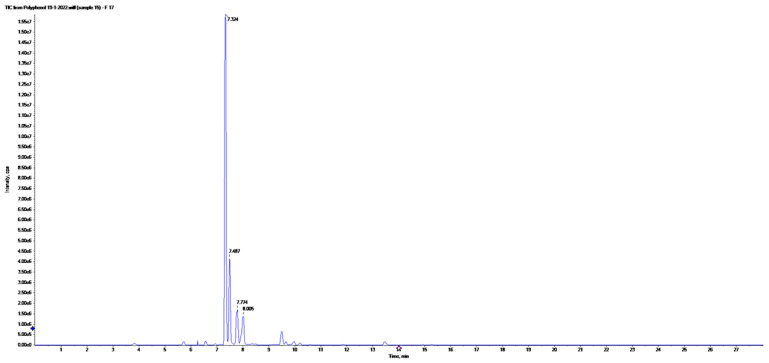
The LC-ESI-MS/MS chromatograms obtained in MRM mode of phenolic and flavonoids of the 70% ethanolic extract of *Maesa indica*.

Liquid chromatography combined with electrospray ionization triple quadrupole mass spectrometry was used to detect and quantify phenolic compounds of the ME. The analysis condition was described (in material and method) and the obtained peaks were identified based on the inserted parent ions (Q1), product ions (Q3) and retention times of the previously injected standard phenolic compounds and reported literatures. The LC-ESI-MS/MS chromatograms obtained using MRM mode for a mixture of 21 standard phenolic and flavonoid compounds are represented in Figure 8. The LC-ESI-MS/MS chromatograms obtained in MRM mode of phenolic and flavonoids of ME are represented in Figure 9. The analysis revealed the identification of fifteen phenolic compounds (chlorogenic acid, gallic acid, caffeic acid, rutin, methyl gallate, coumaric acid, vanillin, naringenin, querectin, ellagic acid, 3.4-dihydroxybenzoic acid, kaempferol, ferulic acid, syringic acid, and luteolin) (Table 2). The major compound was chlorogenic acid at a concentration of 1803.84 μg/g. Quercetin, caffeic acid, rutin, and chlorogenic acid were identified previously in the fruit acetone and methanol extract of *Maesa indica* [5]. Other identified compounds were encountered for the first time in this study. This is the first time investigating the phenolic compounds in *Maesa indica* using LC–ESI-MS-MS- MRM analysis.

### 2.3. Anti−Coronavirus 229E Activities of ME, ZnO NPs and ZnO NPs Combined with ME

Generally, the ability of viruses to multiply and infect can be measured by using suitable cell lines in artificial systems called in vitro antiviral assessments. It is an effective and safe assay as it gives important information about the tissues and the effect of the tested sample on it as a prior step to preclinical and clinical assessments [26]. In traditional medicine, many hundreds of plants, herbs, and marine herbs were used to treat viral infections. The viral resistance against antiviral medications has motivated the researchers to find new, effective, and safe antiviral medications. Plants have many types of phytochemicals, which have antiviral activity against many viruses of both DNA and RNA viruses. About 60% of the market anti-infective medication consists of natural materials. Investigating the plants and herbs for their antiviral activity has led to the discovery of many new antiviral compounds [27]. The genus *Maesa* was reported to have antiviral activity. The aqueous extract of *Maesa lanceolate* Forsskleaves had virucidal effect against herpes simplex virus type 1 (HSV−1) [28]. *Maesa latifolia* Pit. leaves and stem extract were reported to have anti-hepatitis C virus (HCV) activity [29].

Nanoparticles have many uses in modern medicine. They are used for analysis and imaging diagnosis, and treating various diseases in a simple way cannot be done otherwise [30]. ZnO NPs are believed to have strong and safe antiviral activity due to their physical and chemical characteristics. ZnO NPs have been reported to be promising material in producing nano-vaccines against both RNA and DNA viruses. ZnO NPs possess antiviral activity by preventing the virus from entering the cells [22]. 

Coronaviruses (CoVs) are positive, enveloped, and single-stranded RNA viruses. The 229E strain of coronavirus associated with many respiratory illnesses started from the mild common cold to sever pulmonary complications and pneumonia [31]. This virus is one of the seven human infectious coronaviruses that include COVID-19.

In this study, we assessed antiviral activity against coronavirus 229E of ME, green synthesized ZnO nanoparticles, and ME combined with ZnO nanoparticles. The result of the assay revealed that the three samples were suggested to have anti-coronavirus 229E activity with selectivity index (SI) of more than 23, and the highest SI was obtained from the ZnO nanoparticles sample (40.92).

In the assay of anti-viral activity, it is important to measure the cytotoxicity of the investigated sample. CC_50_ (concentration of test sample required to reduce cell viability by 50%) and IC_50_ (50% inhibition concentration) were measured on the three samples, and all of them were suggested to be active against coronavirus 229E (Table 3). The green synthesized ZnO nanoparticle CC_50_ was the highest one by 292.61 ± 0.93 µg/mL while the lowest CC_50_ obtained from plant extracts combined with ZnO nanoparticles was 138.49 ± 0.26 µg/mL. The IC_50_ of the plant extract combined with ZnO nanoparticles was the lowest one by 5.23 ± 0.18 µg/mL while the highest one was produced by a plant extract of 9.97 ± 0.38 µg/mL. On the other hand, ZnO nanoparticle IC_50_ (7.15 ± 0.1 µg/mL) was lower than that produced by a plant extract (9.97 ± 0.38 µg/mL). The three tested samples did not produce any changes to the density or the shape of cells. ZnO nanoparticles’ possible anti-coronavirus activity could be caused by decreasing virus replication and pathogenesis in vivo by binding with viral M2−1 protein, which is involved in pneumovirus replication [22]. Possible ME anti-coronavirus 229E activity could be due to the presence of a high level of phenolic and flavonoid compounds that have the ability to prevent virus replication and pathogenesis by chelating metal ions [32]. This result suggested that ZnO nanoparticles combined with ME could have superior anti-coronavirus 229E activity to ZnO nanoparticles and plant extracts alone while ZnO nanoparticles could have superior anti-coronavirus 229E activity than plant extract. The tested samples could be considered good candidates for further experiments as anti-coronaviruses. We suggest this because previous research claimed that the first virus-to-cell fusion event and the propagation of cell-to-cell infection are both inhibited by flavonoids’ antiviral mode of action [33]. Flavonoids possess a variety of properties, some of the flavonoids of which are suggested to affect the function of the immune system [34]. Flavonoids are reported to prevent the binding and entry of viruses into cells, interfere with the stages of the viral replication mechanism, or translate protein processing that prevents the release of the viruses to reinfect other cells. Based on antiviral mechanisms of action, flavonoids can be prophylactic inhibitors, therapeutic inhibitors, or indirect inhibitors by interacting with the immune system [35]. Another study concluded that flavonoids are evidence-based natural sources of antivirals against coronaviruses and have a potential role in the management of it [36].

The possible anti-coronavirus 229E activity of ME can be also correlated to the identified components as chlorogenic acid (CGA) which has been reported to be a potent coronavirus 229E inhibitor when it is investigated using network pharmacology approach, followed by molecular docking [37]. Quercetin, caffeic acid, and rutin are reported to prevent infection of normal human fibroblast lung cells by coronavirus 229E [38]. Gallic acid is a potent immunostimulant, and therefore, it is considered to be a strong weapon against viruses, especially coronaviruses, through the inactivation of immunosuppressive agents and proliferation of T-cells, B-cells, and neutrophils [39]. Another in silico study using molecular docking, using AutoDock Vina software, confirmed that ellagic acid, *p*-coumaric acid, kaempferol, and quercetin have a strong interaction effect on the coronaviruses’ target enzymes. The assay measured the binding ability against the main protease and RNA-dependent RNA polymerase viral enzymes confirming their inhibition effect on it [40]. Caffeic acid and its derivatives reported to have antiviral activity against COVID-19 and other microbes according to [41]. A docking investigation against COVID-19 using rutin concluded that rutin might be an effective inhibitor of many COVID-19 protein targets, especially the essential protease [42]. Chlorogenic acid (CGA) has been reported to be a potent anti-COVID-19 inhibitor when it is investigated using a network pharmacology approach followed by molecular docking [37]. A previous study confirmed that chlorogenic acid, ferulic acid, and syringic acid have promising molecular docking scores. As we identified these compounds in ME, it could have promising antiviral activity against human coronavirus (229E). Therefore, we recommended further in vivo and clinical studies on the plant extract and its green synthesized zinc oxide nanoparticles as well as their combination to investigate their activity against human coronavirus 229E. [43]

## 3. Materials and Methods

### 3.1. Plant Materials

Aerial parts before blooming of *M. indica* Roxb. A.DC. were obtained from EL−MAZHAR botanical garden, El-Barageel, Giza, Egypt. Therese Labib, senior botanist at El−Orman Botanical Garden and a consultant of plants taxonomy at Ministry of Agriculture, Egypt, preformed verification of the plant. The number of specimen Voucher was (19 June 2022), and it was kept in the herbarium of Pharmacognosy Department of Faculty of Pharmacy, Cairo University. After air-drying the plant material, it was ground into a coarse powder, stored in a suitable amber glass, placed in an airtight container, and stored at room temperature.

### 3.2. Extraction of Plant Materials

Two hundred grams of the dried powdered *M. indica* aerial parts were extracted using 500 mL of 70% ethanol (three times until complete extraction) at room temperature. The ethanol was evaporated using rotavapor until complete dryness, yielding 3.4 gm of thick extract (ME). 

### 3.3. ZnO NPs Green Biosynthesis

ME was used for synthetization of ZnO NPs according to slightly modified Attia et al. method [44]. Double distilled water (500 mL) was used for dissolving zinc acetate (5 gm). ME (500 mg) was dissolved in 50 mL of ethanol then mixed with zinc acetate solution. The mixture was heated for 20 min on a water bath, then the pH was adjusted to 12 using 3−5 drops of ammonium hydroxide (NH_4_OH) and the mixture was left for 45 min to ensure the formation of nano-ZnO. The mixture was centrifuged at 4000 rpm then it was washed twice with doubly distilled water followed by double washing with ethanol. The last step was freeze-drying of the powder leading to the formation of grayish white powder of nano-ZnO.

### 3.4. Description of Nano-ZnO Particles

The analysis of nano-ZnO powder was done using a Shimadzu 1601 (Shimadzu Corporation, Japan) UV-vis spectrometer that adapted at the range between 200 and 600 nm. Attenuated total reflectance (ATR) mode on a Schimadzu FTIR Affinity−1Spectrophoscopy instrument (Kyoto, Japan) was used to analyze the phytochemicals, which led to the formation of nano-ZnO. Particles size and zeta potential of formulated ZnO nano-particles were measured using a Zetasizer (HT Laser, ZEN3600 Malvern Instruments, Malvern, UK). TEM (JEOL−JEM−1011, Tokyo, Japan) was used to measure and identify the size distribution, the shape, and the size of green synthesized nano-ZnO. For sample preparation, 3−5 drops of the suspension of prepared ZnO nanoparticles reacted with carbon film coated on copper TEM−grid, then the sample was stored at 24 °C until complete dryness, then we started ZnO nanoparticles’ photo recording. The sample was examined with CuKα1 X-ray diffractometer radiation (λ = 1.5406 A°). The method was performed at 30 mA with 40 kV and 2θ over the range from 20° to 90°. The morphological description including the shape and the size of ME green synthesized ZnO NPs were investigated by TEM (JEM−1230, JEOL, Tokyo, Japan). The samples were prepared by adding a drop of colloidal ZnO NPs solution to a copper grid coated with an amorphous carbon film and drying the solvent under vacuum for 8 h before loading onto the sample holder. The AMT software was adjusted for nanoparticle size measurements using a digital TEM camera. The fabricated ZnO NPs average size was calculated by measuring over 100 ZnO NPs in at least 20 random locations on the TEM grid in magnified microphotographs.

### 3.5. LC−ESI−MS/MS−MRM Profiling of Polyphenols

ME was analyzed using liquid chromatography–electrospray ionization-tandem mass spectrometry (LC−ESI−MS/MS) to recognize the phenolic and flavonoid compounds in the plant extract. The separation system was carried out using Exion LC AC and SCIEX Triple Quad 5500 + MS/MS, and the ionization was performed using an electrospray ionization (ESI).

#### Multiple-Reaction Monitoring (MRM) Mode

The column used in the analysis was ZORBAX Zorbax Eclipse Plus C18 column (4.6 × 100 mm, 1.8 µm). The separation was done using gradient elution technique as the mobile phase concentration was changed during the chromatographic run. Two mobile phases were used; the first one was formic acid (0.1%) dissolved in water while the second one was LC grade acetonitrile. Before starting the separation, the mobile phase was adjusted as described in Table 4. The assay mobile phase adjusted at 0.8 mL/min and 3 µL of sample was injected to the column and temperature was kept constant at 40 °C during the run. For analysis of certain polyphenolic compounds, multiple-reaction monitoring (MRM) with both negative and positive ionization modes was used during the same run. The parameters of MS analysis were set as:

(1) Capillary temperature of 400 °C;
(2) Ion Spray voltage: 4500 for positive mode and −4500 for negative mode;
(3) Curtain gas: 25 psi;
(4) Nebulizer gas at 55 psi with a declustering potential: 50;
(5) Collision energy: 25;
(6) Collision energy spread: 10.

### 3.6. Antiviral Activity Assay

Antiviral activity was measured on a coronavirus (229E), which is a low pathogenic coronavirus, and on Vero E6 cells that were obtained from Nawah−Scientific, Cairo, Egypt. DMEM medium was used for cultivation of Vero E6 cells, and the media was provided with 10% serum of embryonic bovine and 0.1% of mixture of antibiotic and antimycotic solution. The used materials in this study (trypsin−EDTA, antibiotic/antimycotic mixture, and DMEM medium embryonic bovine serum) were obtained from Gibco BRL.

The antiviral and cytotoxic effect were determined using crystal violet method following the cytopathic inhibition effect (CPE) according to [45]. The activity was measured on African green monkey kidney epithelial cells named (Vero E6). One day before the infection, the Vero E6 cells were cultured at 2 × 104 cells density per well in a 96-well plate. The next day, the media was removed, and all cells were washed using saline buffered with phosphate solution. The crystal violet technique was used to measure coronavirus (229E) infectivity by assaying the cell viability percentage. The Vero E6 cells were infected with coronavirus (229E) using 0.1 mL of 229E suspension that contains CCID50 (1.0 × 106) of virus. This dose was the suitable dose to produce the intended cytopathic inhibition effect in only two days of incubation. The examined samples (0.01 mL) were added to the Vero E6 cells that were infected with 229E coronavirus. The anti-coronavirus activity of the plant extract was measured using 10-times diluted concentration ranging between 0.1 µg/mL and 1000 µg/mL. The negative control sample was the sample containing Vero E6 cells, which were not infected with 229E coronavirus or treated with the examined samples. The positive control sample was the sample containing Vero E6 cells infected with the 229E coronavirus but was not treated with any treatment. During 72 h of incubation at 37 °C in carbon dioxide, CPE was measured using the optical microscope. The Vero E6 cells layer after incubation was washed with phosphate buffered saline (PBS) water and subsequently stained with crystal violet. The dye was prepared by dissolving 0.03% of crystal violet dye in 2% ethyl alcohol mixed with 10% formalin. The cell layer was washed again with PBS, then it was left to dry. The optical density of each well of the assay plate was quantitatively measured using spectrophotometric method at 570/630 nm. The anti-coronavirus activity of each sample was studied using the following equation [46]:

Antiviral activity = [(mean optical density of negative controls − mean optical density of positive controls)/(mean optical density of test−mean optical density of positive controls)] × 100%

The 50% cytopathic effect (CPE) inhibitory dose (IC_50_) was measured depending on the previous results.

The cytotoxicity of the samples was assessed during this study. Vero E6 cells were seeded in a culture plate, each well of the 96 wells contain 2 × 104 cells. After incubation for one day, a serial dilution of the samples was added to the wells followed by complete incubation for another 3 days. After incubation, Vero E6 cells were washed using PBS; after that, the cytotoxicity assay was completed following the same steps as anti−coronavirus activity assay. 

The used software in determination of the 50% inhibitory concentration (IC_50_) in this study was GraphPad PRISM software (San Diego, CA, USA).

## 4. Conclusions

This study was designed to synthesize ZnO NPs using a sustainable method through green biosynthesis using ME. In addition, the phenolic and flavonoid components present in ME were investigated using LC−ESI−MS/MS−MRM analyses for the first time. The antiviral activity against human coronavirus 229E was also studied. The LC−ESI−MS/MS−MRM analysis revealed the qualitative and quantitative determination of fifteen polyphenolic compounds. This study concluded that ME alone, ZnO NPs, and ME combined with green synthesized ZnO NPs could have a promising antiviral activity against coronavirus 229E. ME combined with green synthesized ZnO NPs was the most effective among the tested samples. Further studies are required to fully explore this antiviral potential, which could be beneficial for treating and controlling the spreading of coronavirus 229E. Finally, ME activity against COVID-19 is recommended to be investigated due to its similarity to coronavirus 229E using in vitro and in vivo assays in addition to clinical trials. 

## Figures and Tables

**Table 1:** FTIR spectrum bands of *Maesa indica* 70% ethanolic extract and ZnO NPs synthesized by it.

	ZnO NPs			*Maesa indica* Ethanolic Extract	
No.	The Band	The Corresponding Function Group	No.	The Band	The Corresponding Function Group
1.	3417.86 cm^−1^	O-H group	1.	3417.86 cm^−1^	O-H of alcoholic compound
2.	3934.78 cm^−1^	O-H stretching	2.	2927.94 cm^−1^	O-H of carboxylic acid compounds
3.	Essam M. Abd el kaderi 2283.72 cm^−1^	C-C triple bond of alkynes	3.	2860.79 cm^−1^	O-H of carboxylic acid compounds
4.	1897.96 cm^−1^	C=O stretching of carboxylic group	4.	2430.31 cm^−1^	C-H of alkane group
5.	1801.51 cm^−1^	C=O stretching of carboxylic group	5.	2330.87 m^−1^	
6.	1577.77 cm^−1^	C=C stretching of cyclic alkene	6.	2341.58 m^−1^	
7.	1411.89 cm^−1^	O-H bending of carboxylic group	7.	2167.99 cm^−1^	C-C triple bond of alkynes
8.	1384.89 cm^−1^		8.	1924.96 cm^−1^	C-H bending of aromatic compound
9.	1265.30 cm^−1^	C-O of alcohols and carboxylic acid esters	9.	1870.95 m^−1^	
10.	1184.29 cm^−1^		10.	1635.64 cm^−1^	CH bending and in cycle C-C or stretching of C=O in the phenolic components
11.	1014.56 cm^−1^		11.	1516.05 m^−1^	
12.	887.26	C-H bending	12.	1451.33 m^−1^	
13.	439.77	ZnO NPs band	13.	1384.89 cm^−1^	
14.	416.62 cm^−1^		14.	1269.16 cm^−1^	C-O stretching of aromatic ester compound
			15.	1230.58 cm^−1^	C-O stretching of aromatic ester compound
			16.	1114.86 cm^−1^	C-O of secondary alcohol compound
			17.	1080.14 cm^−1^	
			18.	1053.13 cm^−1^	C-H of alkanes group
			19.	918.12 cm^−1^	=CH bending of an alkene group
			20.	887.265 cm^−1^	
			21.	821.68 cm^−1^	
			22.	775.38 cm^−1^	

**Table 2:** MRM parameters and identification of phenolic compounds in 70% ethanolic extract of *Maesa indica* using LC–ESI-MS/MS.

Name	Conc. (µg/g)	Q1 (*m*/*z*)	Q3 (*m*/*z*)	RT (min)	CE (V)	CXP (V)	DP (V)
Gallic acid	10.81	168.9	124.9	3.9	−30	−11	−110
		168.9	79	3.9	−30	−11	−110
Caffeic acid	33.66	178.9	135	8	−22	−9	−115
		178.9	107	8	−30	−7	−115
Rutin	2.85	609	299.9	9.7	−48	−15	−230
		609	270.9	9.7	−70	−9	−230
Coumaric acid	9.79	162.9	119	9.5	−20	−7	−90
		162.9	93	9.5	−40	−5	−90
Vanillin	18.50	151	136	9.6	−12	−9	−140
		151	92	9.6	−16	−7	−140
Naringenin	31.99	271	151	15	−24	−25	−130
		271	119	15	−34	−11	−130
Querectin	1.54	301	151	13.6	−28	−9	−50
		301	178.8	13.6	−20	−7	−50
Ellagic acid	2.67	301	145	9.9	−40	−14	−120
		301	245	9.9	−38	−14	−120
3.4−Dihydroxybenzoic acid	41.79	152.9	109	5.8	−40	−5	−75
		152.9	90.9	5.8	−20	−7	−75
Hesperetin	ND	301	164	15.6	−23	−10	−125
		301	136	15.6	−38	−10	−125
Myricetin	ND	317	179	11.7	−19	−10	−100
		317	137	11.7	−26	−10	−100
Cinnamic acid	ND	146.9	102.6	14.2	−17	−6	−60
		146.9	77	14.2	−33	−6	−60
Methyl gallate	0.30	183	124	7.5	−30	−10	−110
		183	140	7.5	−30	−10	−110
Kaempferol	5.23	284.7	93	15.3	−46	−10	−120
		284.7	116.8	15.3	−52	−10	−120
Ferulic acid	18.51	192.8	133.9	10.2	−16	−5	−25
		192.8	177.9	10.2	−12	−5	−25
Syringic acid	12.77	196.9	122.8	8.4	−24	−5	−30
		196.9	181.9	8.4	−12	−5	−30
Apigenin	ND	269	151	15	−15	−7	−35
		269	117	15	−15	−7	−35
Catechin	ND	288.8	244.9	7.3	−16	−8	−40
		288.8	109	7.3	−32	−8	−40
Luteolin	8.39	284.7	132.9	13.5	−38	−10	−50
		284.7	150.9	13.5	−26	−10	−50
Chlorogenic acid	1803.84	355.1	163	7.8	21	10	46
		355.1	89	7.8	75	14	46
Daidzein	ND	255.1	199	13.4	28	10	125
		255.1	91.1	13.4	44	10	125

Collision gas (CAD) Collision energy (CE). Collision cell exit potential (CXP) Declustering potential (DP).

**Table 3:** Antiviral activity of 70% ethanolic extract of aerial parts of *Maesa indica*, ZnO NPs, and ZnO NPs combined with 70% ethanolic extract of aerial parts of *Maesa indica* against coronavirus 229E.

The Sample	CC_50_	IC_50_	SI	Unit
ZnO nanoparticles	292.61 ± 0.93	7.15 ± 0.10	40.92	µg/mL
ZnO NPs combined with ME	138.49 ± 0.26	5.23 ± 0.18	26.47	µg/mL
70% ME	235.94 ± 0.32	9.97 ± 0.38	23.66	µg/mL

CC_50_, 50% cytotoxic concentration; ICf_50_, 50% inhibition concentration; SI, selective index. Values are expressed as mean ± S.E.

**Table 4:** The mobile phase program used for LC−ESI−MS/MS−MRM Profiling of Polyphenols of 70% ethanolic extract of aerial parts of *Maesa indica*.

Rate	Holding Time by Min.
2% acetonitrile (LC grade)	0−1 min
2−60% acetonitrile (LC grade)	1−21 min
60% acetonitrile (LC grade)	21−25 min
2% acetonitrile (LC grade)	25.01−28 min

## Data Availability

Not applicable.
